# Functionalized turmeric nanovesicles for precision delivery of doxorubicin in colorectal carcinoma treatment

**DOI:** 10.3389/fphar.2025.1587560

**Published:** 2025-05-30

**Authors:** Chen Meng, Xue Yi, Meitao Duan, Ahmed Mahal, Zhiqiang Zhang, Jungang Ren, Ming Chen, Lin Yang, Moxun Xu, Ahmad J. Obaidullah, Linwei Song, Shuxian Li, Chen Wang

**Affiliations:** ^1^ College of Pharmacy, Jiamusi University, Jiamusi, China; ^2^ Key Laboratory of Functional and Clinical Translational Medicine, Fujian Province University, Xiamen Medical College, Xiamen, China; ^3^ Guangzhou HC Pharmaceutical Co., Ltd, Guangzhou, China; ^4^ Department of Pharmaceutical Chemistry, College of Pharmacy, King Saud University, Riyadh, Saudi Arabia

**Keywords:** turmeric nanocarriers, tumor-homing peptide, drug encapsulation, colorectal carcinoma, biocompatible chemotherapy

## Abstract

Nanoscale vesicles have emerged as promising biocompatible vehicles for precision drug delivery, owing to their inherent therapeutic properties and versatile structural configurations. This study introduces an innovative biomanufacturing strategy utilizing curcumin-extracted nanovesicles (TNVs) conjugated with a cancer-selective peptide and encapsulated with doxorubicin to optimize therapeutic outcomes in colorectal malignancies. TNVs were purified through refined ultracentrifugation protocols, demonstrating uniform saucer-shaped morphology with an average size of 162.42 ± 3.67 nm and stable bilayer architecture dominated by triglyceride (30%) and ceramide (11.8%) constituents. Peptide-mediated surface functionalization substantially improved intracellular internalization efficiency in HCT-116 colon carcinoma models. The engineered TNV-P-D formulation exhibited potent tumoricidal activity (IC_50_ = 54.8 μg/ mL), outperforming both unbound doxorubicin (IC_50_ = 795.2 ng/mL) and nonfunctionalized TNV-DOX counterparts (IC_50_ = 129.7 μg/mL). Cell cycle profiling revealed G1-phase blockade (91.3% G1-phase occupancy), corroborating the platform’s proliferation-inhibiting capacity. In murine CT26. WT xenograft models, TNV-P-D administration achieved significant tumor regression (65% volume reduction, p< 0.001) while preserving hepatobiliary function and demonstrating negligible multiorgan toxicity. These results position peptide-augmented phytovesicles as a multifunctional therapeutic system capable of dual-action tumor targeting and systemic toxicity mitigation in colorectal oncology.

## Graphical Abstract

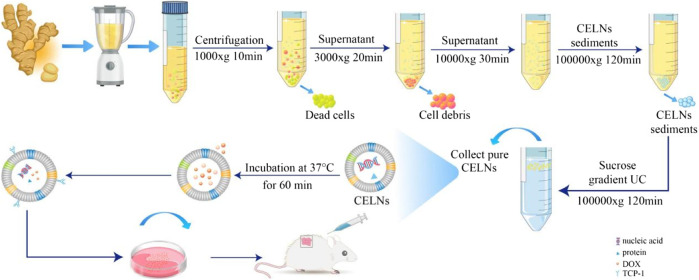

## Introduction

Colorectal malignancy, a life-threatening tumor developing in the gastrointestinal tract, shows epidemiological correlations with nutritional patterns characterized by insufficient fiber consumption and excessive fat ingestion. Currently, the standard treatment for colon cancer in clinical practice is radical tumor resection. Additionally, postoperative combination therapy involving radiotherapy and chemotherapy can decrease the recurrence rate in patients. Prognostic benchmarks for patient cohorts at the half-decade milestone post-surgery is approximately 50–60 percent, primarily due to metastasis and recurrence (Shinji et al., 2022). Colon cancer is a malignancy of the digestive tract that is challenging to detect in its early stages and difficult to excise completely in a timely manner. Radiotherapy and chemotherapy exhibit significant toxicity. The adverse reactions experienced by patients during treatment were significant, and the treatment scale proved challenging to manage. There is an urgent necessity to develop high-efficiency, low-toxicity anti-colon cancer drugs to address this issue.

Plant exosome-like nanovesicles (PEVs) are membranous vesicles secreted by plant cells, ranging in size from 30 to 300 nm. They possess a phospholipid bilayer as their fundamental structure and encapsulate various lipids, proteins, nucleic acids, and other active substances. PEVs exhibit structural and functional similarities to animal exosomes, which serve as messengers for intercellular information exchange and are involved in the physiological processes of various diseases in humans. This material exhibits favorable biocompatibility and low immunogenicity, enabling it to traverse biological barriers while also reflecting the physiological and pathological states of cells (Ambrosone et al., 2023; Cao et al., 2023).

Medicinal plant exosome-like nanovesicles (MPEVs) represent a distinct category of extracellular vesicles (EVs) exclusively sourced from medicinal plants. In contrast to EVs from other origins, MPEVs encapsulate the pharmacodynamic compounds of their respective plants, resulting in enhanced biological activity compared to standard plant-derived EVs. Furthermore, it has shown distinct benefits as a therapeutic agent and drug carrier (Baldassari et al., 2023; Fang and Liu, 2022; Jiang et al., 2024; Jiang et al., 2023; Karamanidou and Tsouknidas, 2021; Liu et al., 2022a; Pomatto et al., 2023; Wang et al., 2013; Zuzarte et al., 2022). MPEVs can be modified beyond encapsulated active ingredients to achieve specific objectives or to produce multi-component synergistic effects (Akbari et al., 2022; Guo et al., 2024).

Numerous studies indicate that MPEVs exhibit anti-tumor, anti-inflammatory, anti-fibrotic, anti-aging activities, liver protection, and hypoglycemic effects (Feng et al., 2023; Gong et al., 2022; Lu et al., 2023; Singh et al., 2020; Takakura et al., 2022; Tan et al., 2022).

Turmeric is a species within the genus *Curcuma* longa L., belonging to the ginger family (Zingiberaceae). It is prevalent in regions such as Fujian, Taiwan, Guangdong, Guangxi, Yunnan, and Tibet. Turmeric is both a medicinal and edible substance, noted in the “Compendium of Materia Medica” for its beneficial therapeutic effects on liver injury (Alvarado-Noguez et al., 2018; Ibrahim al., 2020), as well as its ability to reduce blood pressure and blood lipids concurrently. Turmeric is a frequently utilized medicinal plant among the She ethnic group in Fujian. Curcumin, a natural medicinal and edible compound derived from Curcuma, exhibits a notable inhibitory effect on tumor cells (Nguyen et al., 2020; Wang et al., 2017a; Wang et al., 2017b).

Doxorubicin hydrochloride (DOX) is a chemotherapeutic agent characterized by a wide antitumor spectrum, demonstrating significant antitumor activity against various tumor cells through the inhibition of nucleic acid synthesis in these cells (Jin et al., 2024; Kciuk et al., 2023; Liu et al., 2022b). The synthetic peptide TCPSPFSHC (TCP-1), characterized by its cyclic polypeptide structure and amino acid sequence TCPSPFSHC, exhibits favorable pharmacokinetic properties, including plasma stability and rapid clearance, along with a high affinity for colorectal cancer (Li et al., 2010; Liu et al., 2016a; Liu et al., 2016b). In our exploration of medicinal chemistry and drug discovery, we developed and prepared novel turmeric-derived exosome-like nanovesicles, modified for targeting, and co-incubated them with doxorubicin hydrochloride to investigate their anti-colon cancer effects.

## Materials and methods

### Materials

HCT-116 cells were obtained from the Shanghai Cell Bank. Doxorubicin hydrochloride, PBS buffer, DMEM medium, fetal bovine serum, penicillin-streptomycin bispecific antibody, pancreatic enzyme, and RIPA lysate were procured from Meilun Biotechnology Co., Ltd. Turmeric was obtained from Anhui Bozhou Medicinal Materials Wholesale Center.

### Instrument

Beckman ultracentrifuge (OPTIMA XPN-100); Transmission Electron Microscope (TEM) (Hitachi, HT7700, Japan); Nanoparticle Tracking Analysis (NTA) (Malvern, United Kingdom, Nanosight300); CO_2_ incubator (Sanyo Electric Co., Ltd., MCO-15AC); Tabletop Cryogenic Centrifuge (Kubota, Japan, K21126-F000); Constant Temperature Oscillating Water Tank (Shanghai Jinghong Experimental Equipment Co., Ltd., DKZ-2); High Performance Liquid Chromatograph (Agilent LC1100, United States); HPLC column (Dima Otai Science and Technology Development Center, Beijing Dima, 5 μm C_8_, 150 × 4.6 mm, XDB-C_8_); Microplate Reader (Shanghai Kehua Bioengineering Co., Ltd., Khb ST-360); Infrared Spectrometer (Breuer TENSOR27, Germany).

### Extraction and purification of TNVs

The isolation protocol for curcuminoid nanovesicles commenced with homogenization of rhizome-derived turmeric particulates (1:10 w/v) in ice-cold phosphate-buffered saline (pH 7.4). Following 5-hour hydration at 4°C, the suspension underwent mechanical disintegration through a cell disruptor, succeeded by multilayer filtration to remove macroscopic debris. A sequential centrifugation protocol was implemented: primary clarification at 1,000g (10 min), intermediate pelleting at 3,000g (20 min), and macromolecular elimination at 10,000g (30 min), with progressive supernatant recovery. Ultracentrifugation at 100,000g (2 h) yielded the crude vesicular pellet, which subsequently underwent refined purification via equilibrium density gradient separation. The pellet suspension was stratified using discontinuous sucrose gradients (15-60% w/v) and ultracentrifuged at 100,000g (90 min). The 30-45% sucrose interface fraction, identified as the vesicle-rich zone, was dialyzed against PBS through iterative ultracentrifugation (100,000g, 90 min) to eliminate osmotic stabilizers.The purified nanovesicular suspension (1 mL PBS) underwent cryopreservation at -80°C in light-impermeable vials, with strict avoidance of thermal cycling during storage and handling (Viswanad et al., 2015; Wilson et al., 2020; Zhao et al., 2023).

## TNVs TEM detection

Utilize a pipette to transfer 5 μL of the sample onto the wax tray. Employ a copper mesh to ensure contact between the supporting film and the sample liquid. Allow it to stand for 1 min, then remove the copper mesh. Use a filter paper strip to absorb any excess droplets and lightly dry the surface. Apply a small drop of a 3% phosphotungstic acid solution onto the wax plate. Position the copper mesh containing the adsorbed sample on the dye solution’s surface, ensuring contact between the sample and the solution, and allow it to stand for 1 min. Remove the copper mesh, absorb the excess droplets using a filter paper strip, dry the sample, and capture images using Transmission Electron Microscopy.

### Analysis of TNVs particle size by nanoparticle tracking

Isolated TNVs samples were diluted with 1 × PBS buffer, and the particle size and concentration of TNVs were measured using nanoparticle tracking Analyzer (NTA) with the ZetaView system, calibrated with 110 nm polystyrene particles, and maintained at approximately 23°C and 37°C.

### Lipidomic analysis of TNVs

150 μL of TNVs stock solution was combined with 750 μL of methyl tert-butyl ether, 300 μL of methanol, 225 μL of secondary water, and 10,000 g. The mixture was centrifuged for 8 min to isolate the supernatant. Evaporate the solvent in the supernatant using nitrogen gas. Introduce 300 μL of methanol/isopropanol (1:1, v/v) to re-dissolve the TNVs, followed by centrifugation at 12,000 g for 20 min to collect the supernatant. The supernatant samples that underwent pretreatment were analyzed for the lipid composition of TNVs utilizing the Xevo G2 XS QTof.

### Determination of protein concentration in TNVs

The TNVs suspension underwent sequential extraction cycles, each combined with a defined volume of protein lysis buffer. Following ice-cold homogenization via vortexing for 10 minutes, the lysate was centrifuged (12,000 × g, 4°C, 5 min) to isolate the clarified supernatant. Aliquots (25 μL) of both calibration standards and experimental samples were dispensed into a 96-well microplate, followed by precise addition of 200 μL BCA detection reagent to each cavity. The plate was subjected to orbital shaking to ensure homogeneity prior to a 30-minute thermal incubation at 37°C

### TNVs modification and loading DOX

The TNVs were incubated with TCP-1 at 37°C at a 20:1 (m/m) ratio for 1 h, followed by centrifugation at 100,000 g for 30 min. The resulting TNV-P was then incubated with DOX at 37°C and 150 rpm at a 1:1 (m/m) ratio for 2 h, centrifuged again at 100,000 × g for 30 min, and resuspended to yield TNV-P-D. Drug loading was determined using high-performance liquid chromatography.

### CCK-8 assay

HCT-116 cells at exponential proliferation phase were plated in 96-well microplates (2 × 10^3^ cells/well). Upon achieving 80% confluency, TNV suspensions (100 μL/well) were administered across a 0.1-1.0 mg/mL concentration gradient (incremental steps: 0.1, 0.2, 0.3, 0.4, 0.5, 0.6, 0.7, 0.8, 0.9, and 1 mg/mL). Following 24-hour exposure, 100 μL fresh growth medium supplemented with 10 μL CCK-8 detection solution was introduced to each chamber and maintained at 37°C for 90 minutes. Absorbance quantification at 450 nm was performed using a multimode plate reader. Experimental design incorporated tripartite groupings: negative controls, treatment cohorts, and background blanks (n=5 biological replicates). Cell viability percentages were derived from arithmetic mean ± standard deviation calculations, with half-maximal inhibitory concentrations (IC50) determined through four-parameter logistic regression modeling.

### Cell uptake experiments

The conventional propidium iodide (PI) staining technique was employed to assess cell cycle progression and apoptosis. Following staining of cellular DNA with propidium iodide, the DNA content within the cells was measured via flow cytometry. Subsequently, cell cycle analysis was conducted based on the distribution of DNA content.

### Cell cycle study

The conventional propidium iodide (PI) staining method was used to analyze the cell cycle and apoptosis. After the DNA in the cells is stained with propidium iodide, the DNA content of the cells is determined using flow cytometry, and then the cell cycle analysis is performed based on the distribution of DNA content.

### *In vivo* experiments in animals

A total of 35 male BALB/c mice, aged 6–8 weeks, were selected for *in vivo* measurement of tumor growth. CT26.WT cells (1 × 10^6^) were administered via right subcutaneous injection and categorized into the following groups: blank group, control group, DOX group, TNVs group, TNV-D group, TNV-P group, and TNV-P-D group. After 7 days, tumor size was assessed, revealing a tumor volume of 50 mm^3^ following the initiation of treatment. The protein concentration for each sample was 200 μg, with a dosage of 2.5 mg/kg in the DOX group, while the control group received PBS treatment. Mice are administered doses continuously over a 7-day period, during which their body weights are recorded. Following the conclusion of administration, tumor size in each group and the drug distribution in mice were investigated.

### Data analysis

Statistical comparisons between experimental groups were performed employing parametric tests (one-way ANOVA or twosample Student’s t-tests) through GraphPad Prism 9.5 analytical suite. Quantitative significance thresholds were established with α = 0.05 as the cutoff for rejecting null hypotheses. All experimental results are expressed as arithmetic averages with standard deviations (SD) derived from three biological replicates. Asteriskbased notation indicates statistical magnitude: * (p < 0.05), ** (p <0.01), and *** (p < 0.001) demarcate progressively stringent confidence levels.

## Results

### Extraction, identification, modification and drug loading of TNVs

**FIGURE 1:**
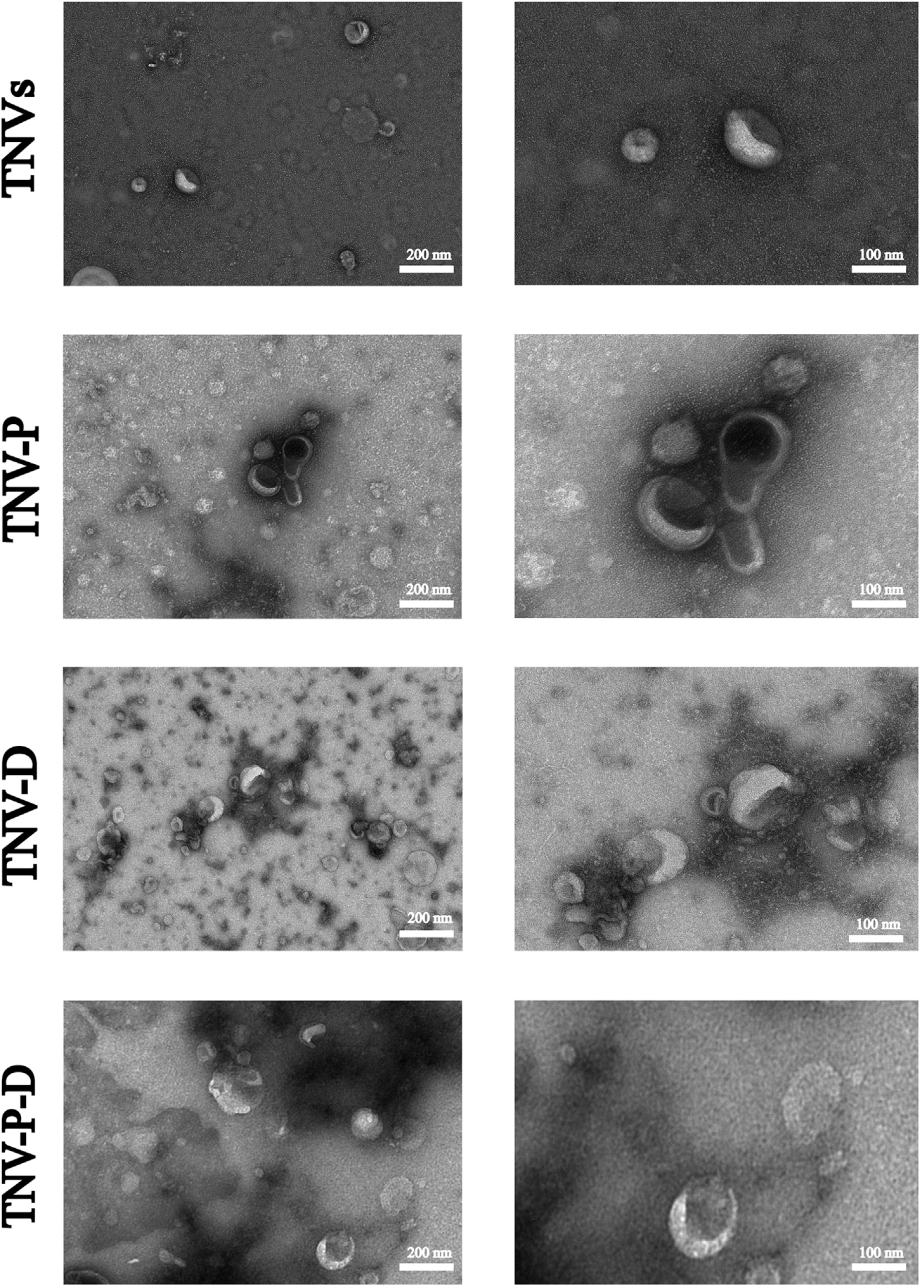
A Transmission electron microscopy of TNVs,TVN-P,TNV-D,TVN-P-D.

**FIGURE 2:**
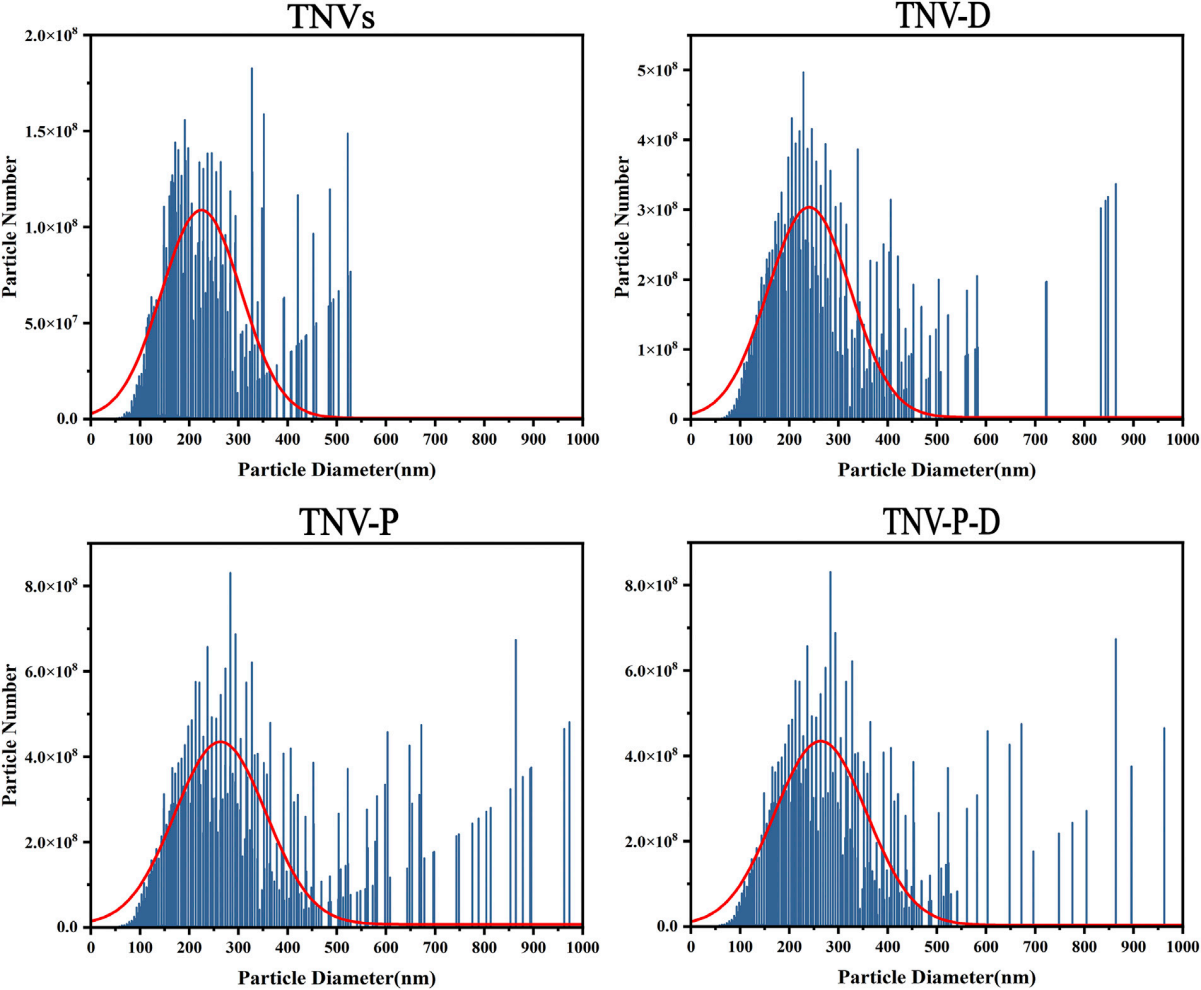
NTA particle size distribution map of TNVs,TVN-P,TNV-D,TVN-P-D.

Figure 1 shows the high and low magnification morphologies of TNVs, TNV-P, TNV-D, and TNV-P-D under TEM. It can be observed that there may be some differences in morphology among TNVs, TNV-P, TNV-D, and TNV-P-D, but all exhibit a distinct saucer shape double-layer membrane structure.The particle sizes of vesicles measured by NTA predominantly ranged from 162.42 ± 3.67 nm (Figure 2), aligning closely with the dimensions of plant extracellular vesicles reported in prior research. The zeta potential is measured at - 25.71 ± 0.38 mV, suggesting that it exhibits good stability. The morphology and size conform to the definitional criteria for extracellular vesicles in plants.During the construction process of the TNV-P-D complex, the particle sizes and zeta potentials of TNV-P, TNV-D, and TNV-P-D were examined respectively. The measurements revealed that TNV-P had a particle size of 186.06 ± 4.98 nm and a zeta potential of −23.61 ± 0.41 mV. For TNV-D, the particle size was 168.15 ± 3.66 nm, with a zeta potential of −20.68 ± 0.41 mV. Meanwhile, TNV-P-D exhibited a particle size of 188.01 ± 4.31 nm and a zeta potential of −17.27 ± 1.03 mV

**FIGURE 3:**
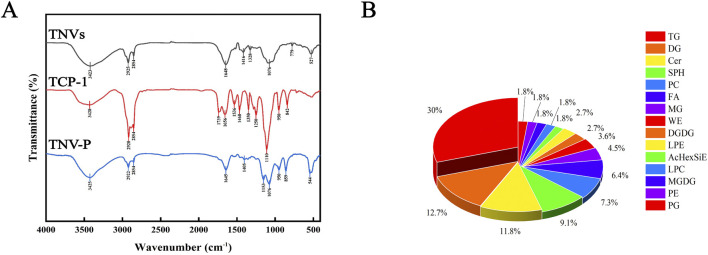
**(A)** Infrared pattern of TNVs; **(B)** Pie chart of lipid composition ratios.

After the TNVs modified by TCP-1 were lyophilized, the infrared spectra of TNV-P demonstrated the successful ligation of plant vesicles (phospholipid bilayers) and TCP-1, demonstrating the co-existence of TNVs and TCP-1 characteristic peaks (Figure 3A). The kit measured the protein concentration of TNVs at 5.3 mg/mL. These distinctive peaks show that TNVs and TCP-1 chemically link to produce a stable complex that supports the development of targets for plant vesicle alteration (TNV-P). According to liquid chromatography, TNVs have a 23% drug loading capacity.

According to studies, lipids play a significant role in the vesicle phospholipid bilayer and their effectiveness is inextricably linked to the function of certain lipid components. Lipidomic study of TNVs is therefore required. As illustrated in Figure 3B, up to six lipid components - triglycerides (TG, 30%), diglycerides (DG, 12.7%), ceramides (Cer, 11.8%), sphingosine (SPH, 9.1%), phosphatidylcholine (PC, 7.3%), and fatty acids (FA, 6.4%)—were discovered to make up more than 5%. The extraction of TNVs was successful since the extraction’s proportion and composition matched those of plant exosome-like nanovesicles.

### Cell viability was determined by CCK-8 method

**FIGURE 4:**
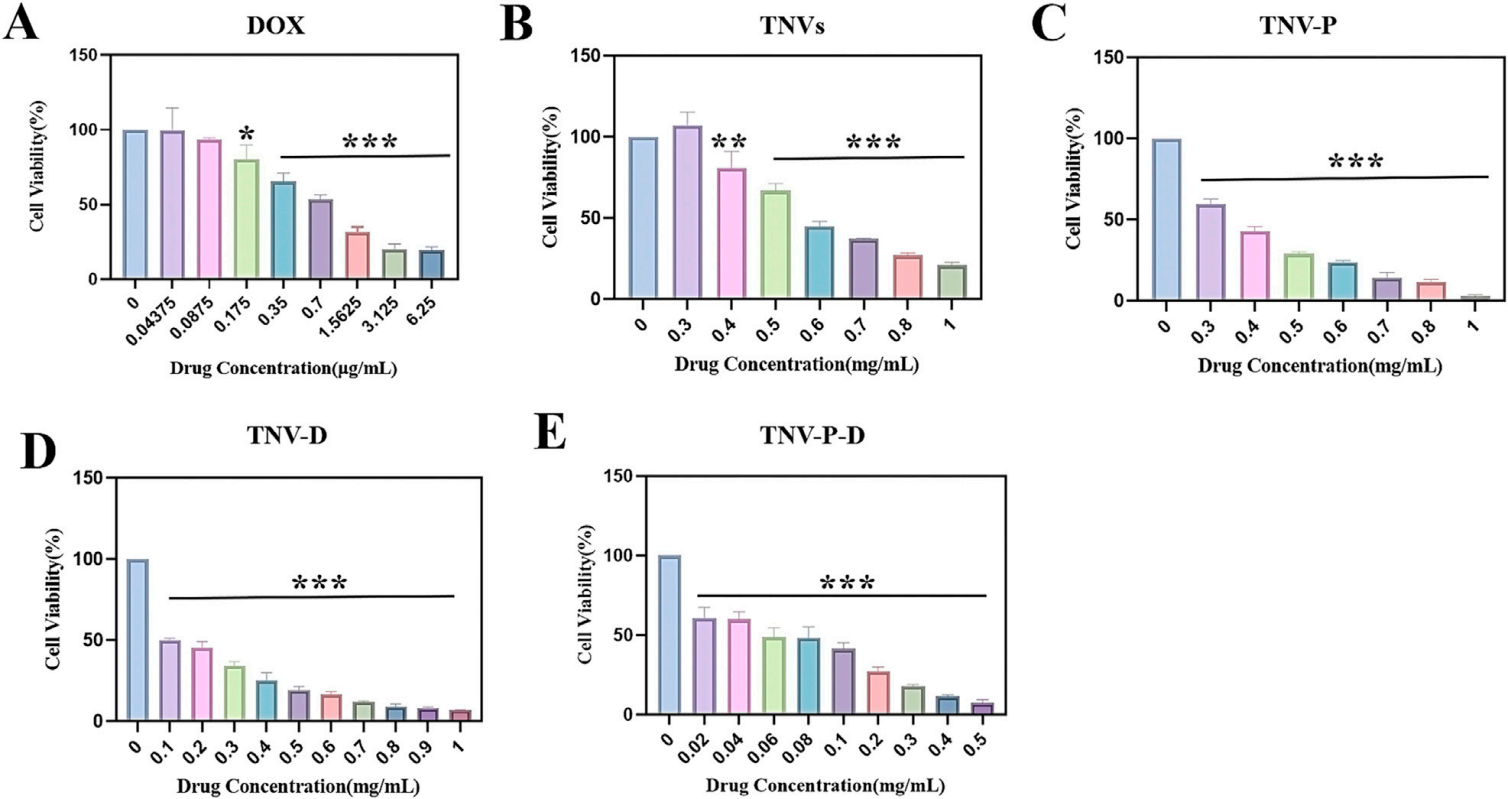
Histogram of cytotoxicity at each concentration of **(A)** DOX; **(B)** Histogram of cytotoxicity of each concentration of TNVs; **(C)** Histogram of cytotoxicity of each concentration of TNV-P; **(D)** Histograms of cytotoxicity at each concentration of TNV-D **(E)**; Histogram of cytotoxicity at each concentration of TNVs-P-D (Blank group VS Administration group *p < 0.05, **p < 0.01, ***p < 0.001) All values were expressed as mean ± SD (n = 3).

The toxicity test of HCT-116 cells is displayed in Figure 4 with the following IC_50_ values (DOX): 793.6 ng/mL (TNVs): 608.9 μg/mL (TNV-P): 355.8 μg/mL (TNV-D): 130.9 μg/mL, and (TNV-P-D): 55.1 μg/mL are included in Figures 4A,B,E, respectively. Cell proliferation was suppressed by the five sample groups, and at the same concentration, TNVs, TNV-P, TNV-D, and TNV -P-D’s capacity to suppress HCT-116 cell proliferation was progressively increased. TNVs and TNVs-P at the same concentration, TNV-P had more ability to inhibit the proliferation of HCT-116 cells, indicating superior targeting of target-modified TNVs.TNV-D and TNV-P-D at the same concentrations, TNV-P-D was more able to inhibit the proliferation of HCT-116 cells. In conclusion, the TNVs-TCP-1-DOX system is more effective at preventing the growth of HCT-116 cells and detecting cancer.

When the DOX concentration was 175 ng/mL, there was a significant difference with the control group (p ≤ 0.05); when the concentration was 350 ng/mL, there was a highly significant difference with the control group (p ≤ 0.001). There was a highly significant difference with the control group (p ≤ 0.01) when the concentration of TNVs was 0.4 mg/mL, and a highly significant difference with the control group (p ≤ 0.001) when the concentration was higher than 0.4 mg/mL. There was a highly significant difference (p ≤ 0.001) between the TNV-P concentration and the control group when it exceeded 0.3 mg/mL. There was a highly significant difference (p ≤ 0.001) between the TNV-D concentration and the control group when it exceeded 0.1 mg/mL. A highly significant difference (p ≤ 0.001) was seen when the concentration of TNV-P-D exceeded 0.02 mg/mL when compared to the control group.

### Cell uptake experiments

**FIGURE 5:**
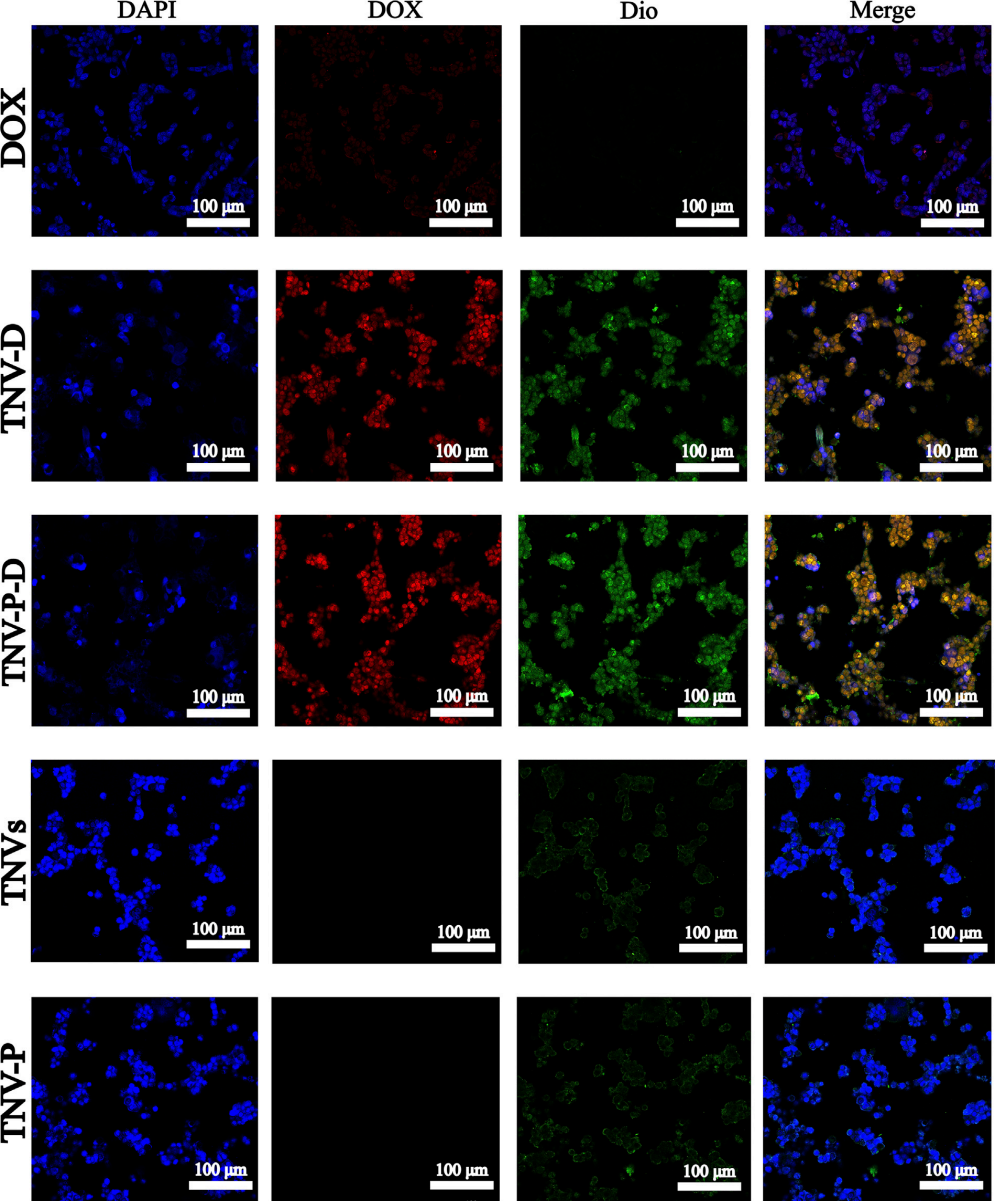
A Uptake of DOX cells; B TNV-D cell uptake map; C TNV-P-D cell uptake map; D TNVs cell uptake; E TNV-P cell uptake map.

**FIGURE 6:**
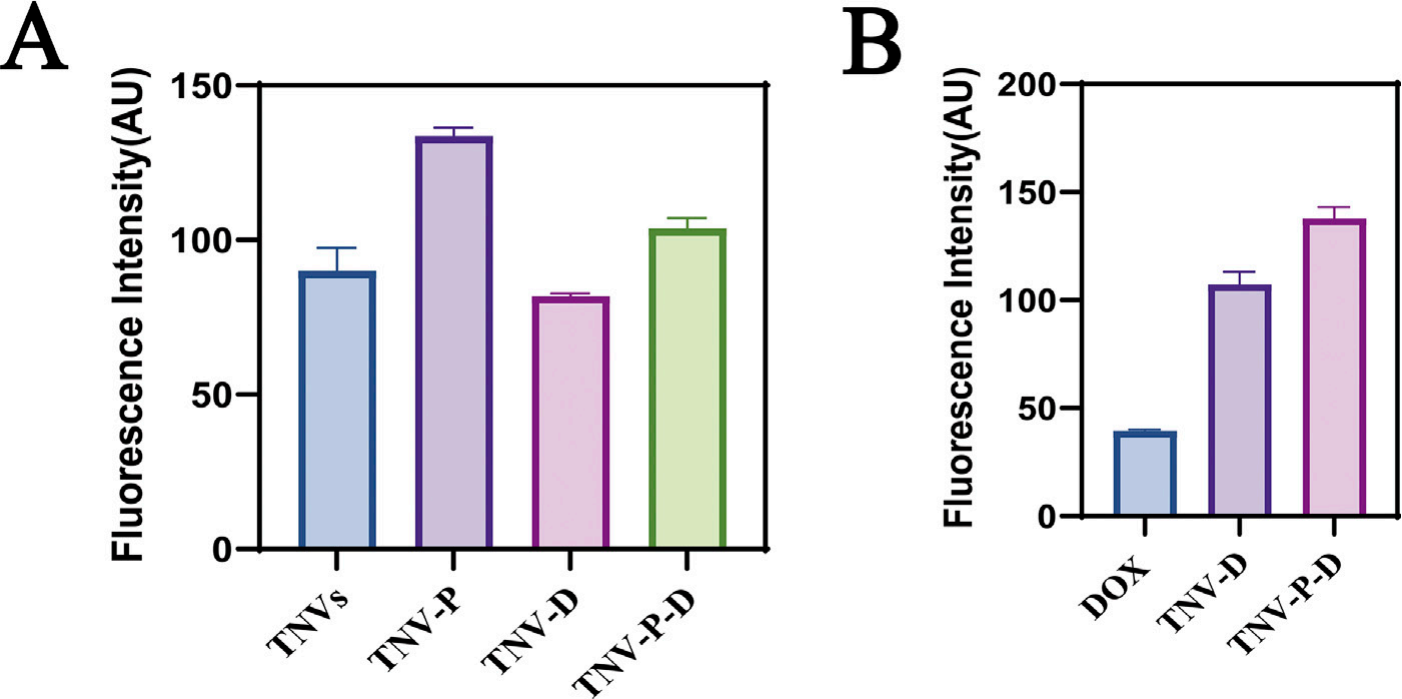
**(A)** green fluorescence intensity; **(B)** red fluorescence intensity.

The uptake of HCT-116 cells in five sample groups was observed using laser confocal microscopy to further illustrate the impact of the constructed targeted drug delivery system TNV-P-D (Figure 5). Red and green fluorescence on the nucleus is seen after the cells have been treated with various medications. Quantifying the fluorescence intensity of each group revealed that TNV-P-D exhibited the brightest red and green fluorescence (Figure 6), while HCT-116 cells had varied degrees of absorption of DOX, TNVs, TNV-P, TNV-D, and TNV-P-D.

### Flow cytometry to detect cell cycle

**FIGURE 7:**
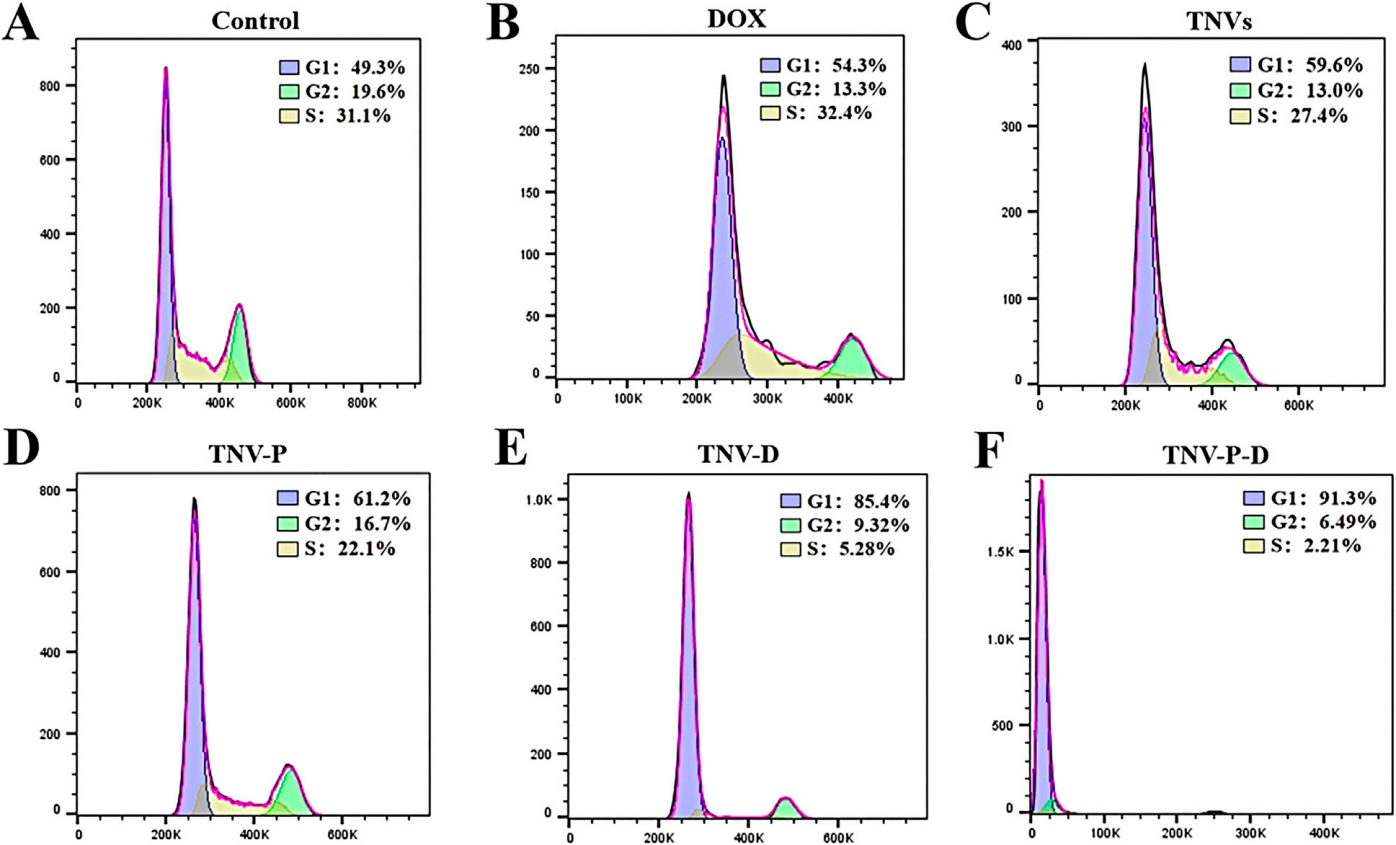
**(A)** Control cell cycle distribution; **(B)** DOX cell cycle distribution; **(C)** TNVs cell cycle distribution; **(D)** TNV-P cell cycle distribution; **(E)** TNV-D cell cycle distribution; **(F)** Cell cycle distribution of TNV-P-D.

Figure 7 illustrates the impact of 24-hour treatment on the cell cycle progression of HCT-116 cells. Across the five treated groups, a significant increase in the G1 phase population was observed relative to the untreated control. The G2 phase cell percentage spanned from 6.49% to 19.6%, whereas the S phase cell percentage fluctuated between 2.21% and 32.4%. These findings are consistent with the well-documented link between proliferation inhibition and cell cycle disruption. The TNV-P-D system demonstrated the most potent capacity to induce G1 phase arrest, effectively halting the cell cycle and curbing cancer cell proliferation.Figure 7 also highlights the changes in the cell cycle of HCT-116 cells after 24 hours of treatment. Compared to the untreated group, a marked elevation in the proportion of cells in the G1 phase was noted across all five treatment groups. The percentage of cells in the G2 phase varied within the range of 6.49% to 19.6%, while the S phase cell percentage oscillated between 2.21% and 32.4%. This trend is commonly associated with the inhibition of cell proliferation and subsequent cell cycle alterations. The TNV-P-D system exhibited the most significant influence on promoting G1 phase accumulation, thereby effectively blocking the cell cycle and impeding cancer cell growth.

### *In vivo* experiments in animals

**FIGURE 8:**
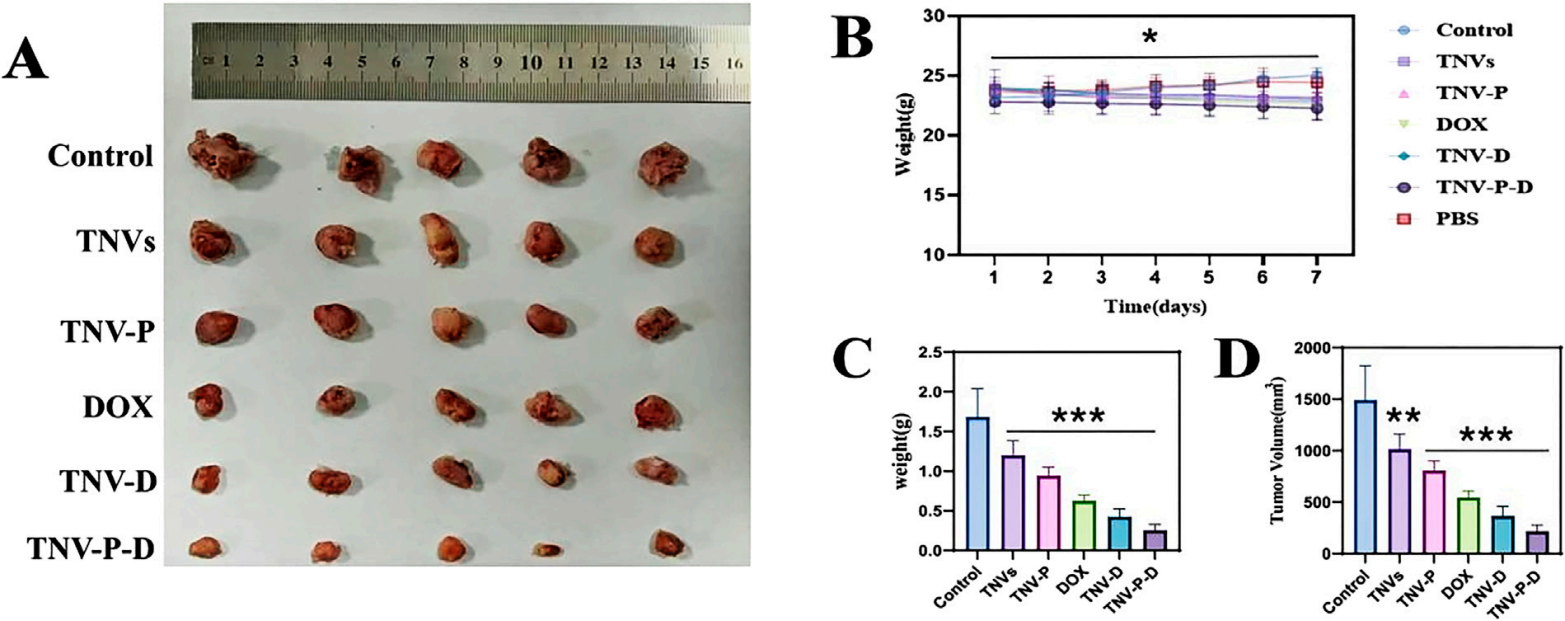
**(A)** Tumor size between groups after the end of treatment; **(B)** Changes in body weight of mice during administration; **(C)** Change in tumor volume in each group; **(D)** Change in tumor weight in each group (Blank group VS Administration group *p < 0.05,**p < 0.01,***p < 0.001) All values were expressed as mean ± SD (n = 3).

When tumor-bearing mice were given tail vein injections (Figure 8A), the tumor tissue volume and weight of each group were compared after 7 days of treatment. The findings demonstrated that the group that received TNV-P-D tail vein injections had significantly lower tumor tissue volume and weight. Body weight was unaffected by the dose for 7 days in a row, as Figure 8B demonstrated. In Figure 8C, the administered group exhibited highly significant differences in tumor size and volume compared to the blank group (p ≤ 0.001). Additionally, the TNVs group demonstrated a significant difference in tumor weight relative to the blank group (p ≤ 0.01). Similarly, the other four groups also showed highly significant differences from the blank group (p ≤ 0.001), as illustrated in Figure 8D. Significant disparities were observed between the administration group and the blank group (p ≤ 0.05). As depicted in Figure 8C, the differences in tumor size and volume between the administered group and the blank group were extremely significant (p ≤ 0.001). In Figure 8D, the tumor weight of the TNVs group was significantly different from that of the blank group (p ≤ 0.01), and the other four groups also exhibited highly significant differences compared to the blank group (p ≤ 0.001).

**FIGURE 9:**
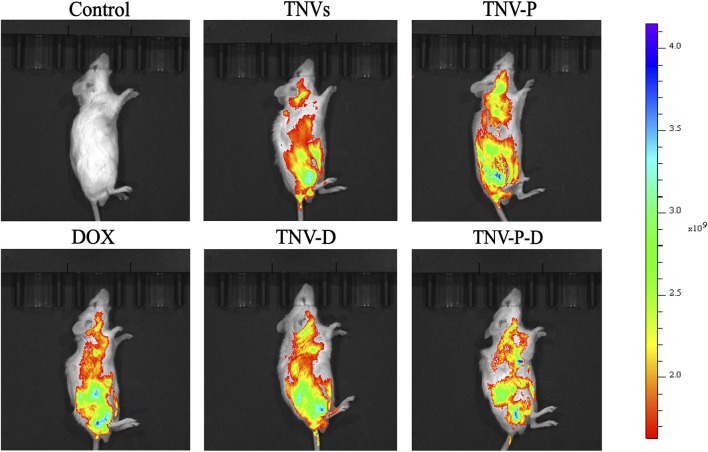
*In vivo* imaging of small animals was used to detect the drug distribution of tumor-bearing mice after tail vein injection for 24 h.

**FIGURE 10:**
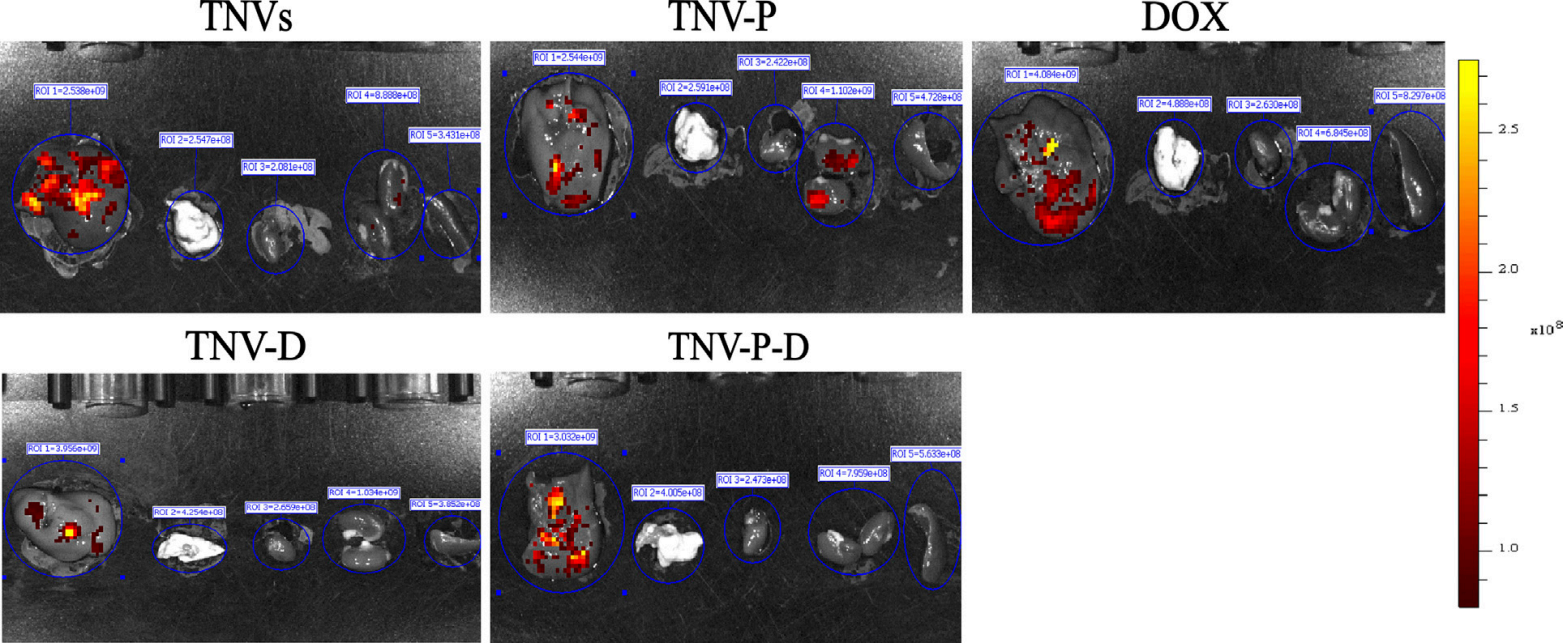
Drug was distributed within the organ within 8 hours of administration.

The ability of TCP-1-modified TNVs to actively target and aggregate at tumor locations was demonstrated by *in vivo* imaging of small animals. As seen in Figure 9, imaging of individual organs reveals that the majority of medications are dispersed throughout the liver (Figure 10).

**FIGURE 11:**
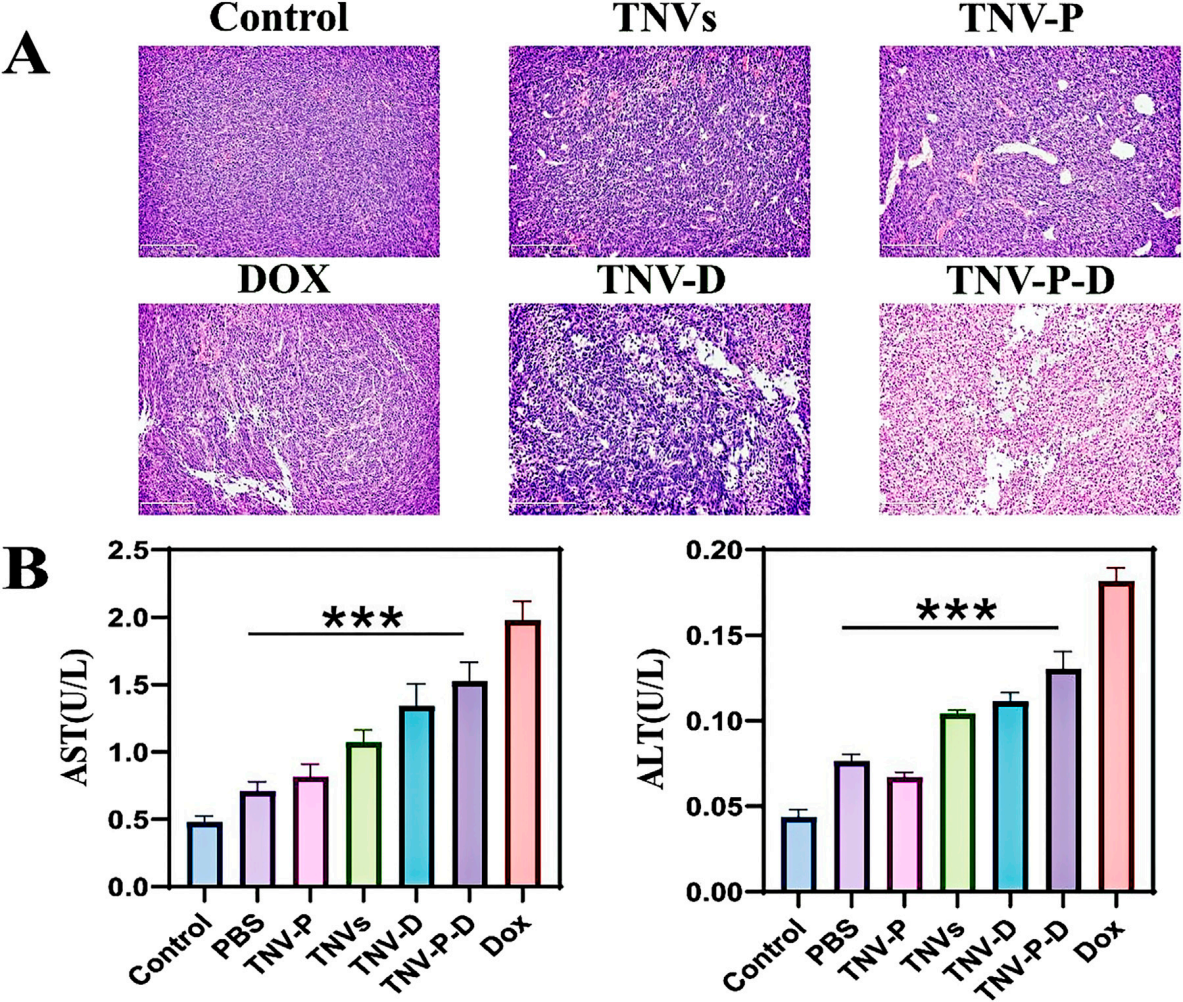
**(A)** HE staining was used to detect the structural changes of tumor tissues after treatment with tumor-bearing mice; **(B)** Biochemical experiments were performed to detect the changes in liver function after treatment of tumor-bearing mice (AST) and (ALT) (Blank group VS Administration group *p < 0.05,**p < 0.01,***p < 0.001) All values were expressed as mean ± SD (n = 3).

**FIGURE 12:**
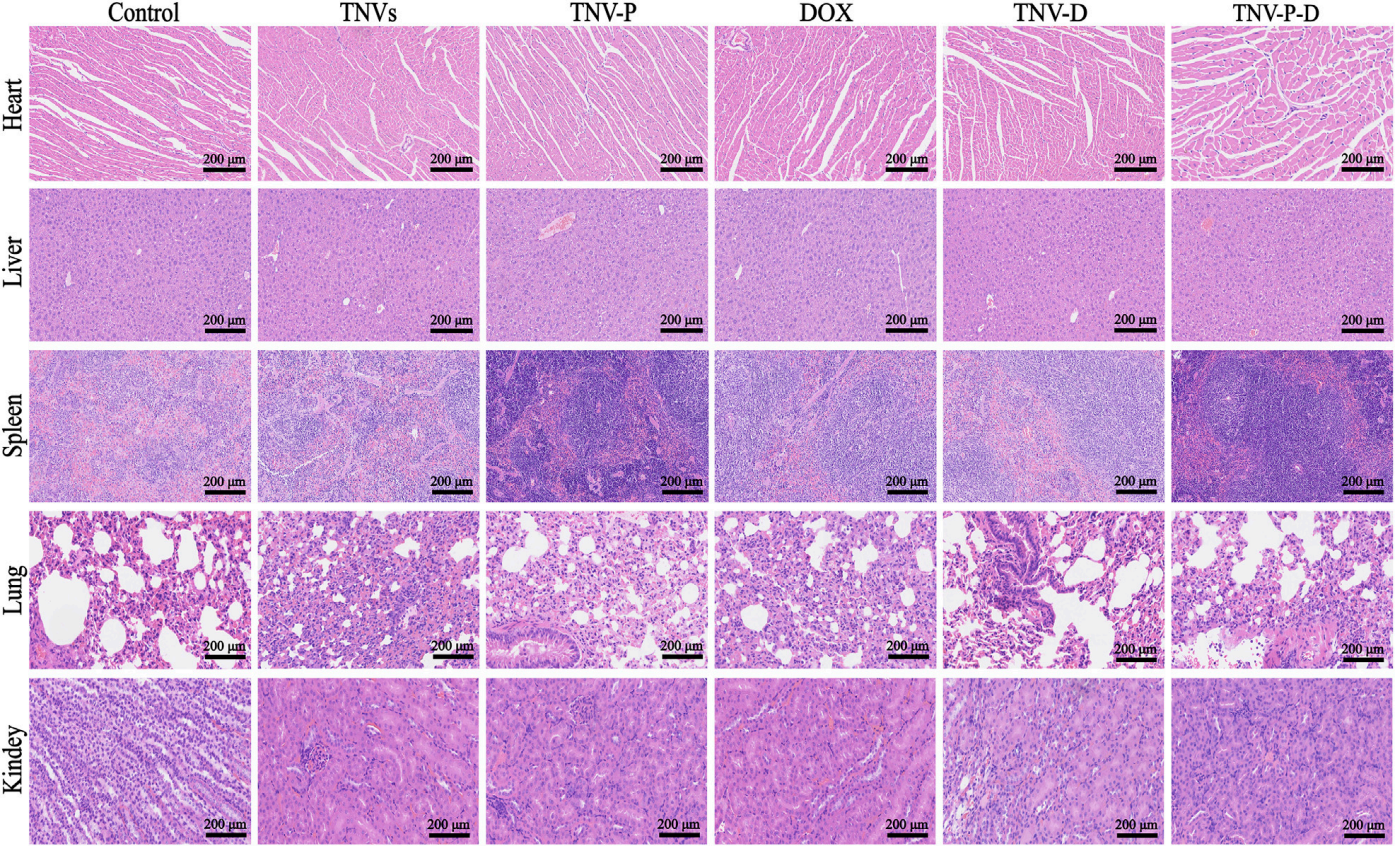
HE staining of major organs.

To assess the safety of TCP-1 modified TNVs and DOX loading in therapeutic applications. At the conclusion of the treatment, the primary organs of the mice—heart, liver, spleen, lungs, and kidneys—along with serum, were gathered for a safety evaluation. The findings from HE staining indicated that the DOX group, TNVs group, TNV-P group, TNV-D group, and TNV-P-D group did not significantly impact the histopathological structure of the heart, liver, spleen, lung, kidney, and other major tissues and organs in mice (Figure 12).Furthermore, serum testing indicated that the treatment across the five intervention groups did not produce a significant impact on the liver function indexes AST and ALT in mice (Figure 11B).

There were highly significant differences between the administration group and the blank group (p ≤ 0.001).

The HE staining results indicated that, in contrast to the PBS treatment group, the TNV-P-D treatment group exhibited significant necrosis, vacuolar changes in cells, and infiltration of inflammatory cells (Figure 11A).

## Discussion

The experimental results indicate that the extracellular vesicle delivery system of turmeric effectively inhibits the proliferation of cancer cells. TCP-1-modified TNVs demonstrated superiority over unmodified TNVs,The modified TNVs can specifically recognize and bind to specific molecules or structures in tumor cells or the tumor microenvironment, thereby increasing the concentration of the drug at the tumor site, enhancing therapeutic effects, and simultaneously reducing damage to normal tissues. In this study, the role of peptides in tumor targeting is to guide drug carriers such as nanovesicles more precisely to the tumor tissue by specifically binding to specific receptors or molecules on the surface of tumor cells, thereby improving the efficiency of drug delivery and treatment. The TNV-P-D system significantly inhibited the proliferation of HCT-116 cells, accelerated the cell cycle, and increased the number of cells in the G1 phase. *In vivo* experiments demonstrated that the system exhibited enhanced tumor targeting and decreased toxicity of DOX, resulting in a safer profile with no significant toxic side effects. The effective biosafety and tumor targeting establish a novel scientific foundation for the advancement of drug delivery systems utilizing plant extracellular vesicles as carriers.

## Data Availability

The raw data supporting the conclusions of this article will be made available by the authors, without undue reservation.
